# Scientific Collaboration at National Institute of the Atlantic Forest (Brazil) on Scopus Database: Analysis of Institutional Domain

**DOI:** 10.3389/frma.2020.601442

**Published:** 2020-12-17

**Authors:** Juliana Lazzarotto Freitas, Fabio Sampaio Rosas

**Affiliations:** ^1^ National Institute of the Atlantic Forest (INMA), Santa Teresa, Brazil; ^2^ College of Agricultural and Technological Sciences, São Paulo State University (Unesp), Dracena, Brazil

**Keywords:** scientific collaboration, co-authorship analysis, domain analysis, institutional identity, National Institute of the Atlantic Forest, professor Mello Leitão Museum of Biology, Brazil

## Abstract

Domain analysis by means of scientific collaboration enables evidencing aspects that are involved in the establishment of relationships between researchers and institutions, such as the influence of institutional management models for the development of collaborative networks. This article aims to analyze the domain through the scientific collaboration network of the National Institute of the Atlantic Forest (INMA), a research unit currently affiliated to the Brazilian Ministry of Science, Technology and Innovation (MCTI), formerly known as the Professor Mello Leitão Museum of Biology (MBML), in order to acknowledge the institutional research identity in its historical journey as a public institution. It is thus analyzed how co-authorship constitutes this network and what research profile it reveals. Co-authorship analysis is adopted as a methodology, as well as the analysis of administrative documents with the survey and categorization of employees, regarding their types of ties to the institution, combined with searches in the Scopus database for the corroboration of institutional affiliations. A corpus of 138 articles published by 41 researchers from 1993 to 2019 is consolidated in this base, which represents 44% of the Institute’s total research collaborators (93 collaborators). Of these 41, 92.5% have temporary links, such as scholarship holders and/or volunteers, with the remaining being public workers. It is recognized that the citation impact of the scientific production of scholarship holders, consigned to the Institute, is less than the citation impact of the volunteers' and public workers' production. It is evidenced that eight of the ten publications with the greatest impact and thematic prominence correspond to the field of zoology, with emphasis on the fields of herpetology and primatology. Macro-level collaborative relations are more intense with the United States, in both areas mentioned, covering 16% of the total corpus of articles in cooperation with that country. Zoology, besides its greater impact, accounts for more than half of the corpus production (65.9%).On the other hand, botany is responsible for 30.4% of the corpus, with its dispersed international cooperation in a broad variety of countries. Individual authorship articles are 57% consigned to botany. In summary, the accomplished analysis will contribute to the development of institutional domain analysis methodologies that present scientific collaboration as a basic procedure.

## Introduction

Studying the field of scientific collaboration involves highlighting social, historical and political aspects that impact the development of relationships between researchers, institutions and countries. Contemporary research on the subject implies the understanding of a variety of concepts such as a distinction between co-authorship practices and other methods of scientific collaboration. The methodologies employed in the field of metric studies of information have not yet been able to accurately measure the relevance and impact that the contribution relationships that are established in a research project can exert in certain contexts, especially when the representative scientific production of an institution derives from the research conducted by researchers (collaborators) with temporary ties. Nevertheless, when metric studies of information are combined with other analytical perspectives such as domain analysis, the elements that portray the identity of a domain can be better grasped.

This article aims to analyze the domain through the scientific collaboration network of the National Institute of the Atlantic Forest (INMA), a research unit currently affiliated to the Brazilian Ministry of Science, Technology and Innovation (MCTI), formerly known as the Professor Mello Leitão Museum of Biology (MBML), in order to acknowledge the institutional research identity in its historical journey as a public institution.

The collaborative networks that contribute to the development of scientific research in an institution can be mapped both inside and outside the organization. This is the case of co-authorship relations within the INMA. This article examines how the collaborative scientific network by co-authorship at INMA is formed and what profile and institutional research identity it reveals.

It is noteworthy that the analysis of the Institute’s collaborative research relationships, by means of co-authorship is of great relevance because it allows the recognition of its main collaborators, since the Institute went from private to federal public administration. These collaborative relationships, defined by the affiliations of its researchers, although stemming from temporary ties, contribute to the constitution of the scientific memory of this institutional domain and they can assist in making decisions regarding the management of scientific policies related to the Institute and the conservation of the Atlantic Forest.

It is worth mentioning that INMA does not present a permanent group of researchers. The science produced by INMA stems from agents with temporary ties, however not less important for the institutional memory characterization. Therefore, co-authorship analysis can be considered as an important element for this end.

The motivation for this study arises from the need to identify who the INMA research agents are and how the research which is associated with the Institute is built in the contemporary science context, where institutional management models influence the collaborative nature of research and, more than this, affect the ways of understanding and mapping institutional collaborative relationships, which may interfere with the impact and visibility of these research.

It is also intended, in the scope of Information Science, to contribute to the improvement of scientific collaboration analysis methodologies that are aimed at the consolidation of institutional domains, in order to broaden the reflection on the co-authorship indicator as an instrument that helps in the establishment of institutional identities and in strategic decisions by research unit managers.

The bibliometric analysis dimension of this institutional domain is emphasized in this article, as it can contribute to in-depth analysis from a historical perspective, thus allowing the revival of the latent memories of actors and partnerships that have been contributing to the restructuring process of INMA as a scientific research unit and to the future institutional aspiration for a position of national and international recognition on the Atlantic Forest research.

Scientific collaboration as a procedure for a domain analysis is a validated practice in previous studies such as Rosas and Gracio (2015) and it can be applied in various contexts. Metric studies of information, as an analytical dimension, can portray scientific domain profiles, by means of production, collaboration and scientific impact indicators. In agreement with Rosas and Gracio (2015) when a bibliometric perspective is combined with other analysis dimensions, it contributes to a wide diagnosis of the science produced, at micro, meso or macro level and it leads to a more accurate approximation of a domain reality.

As a possible complementary analytical dimension to bibliometric analysis, the historical approach proposed by Hjørland (2002) is referred to as a means of deepening a domain analysis, a methodology resource also proposed by the author, in co-authorship with Hjørland and Albrechtsen (1995) in the area of Information Science. A domain is considered to be a narrative that reveals its own behavioral and referential specificities, and may derive from a theme, specialty, discipline, area of knowledge or research institution (Rosas and Grácio, 2015).

Hence, an institutional domain in the scope of research is characterized by scientific collaboration relationships and by the resulting publications of such collaborative relationships. The portrait of these relations and products can be regarded as elements of institutional memory and, in some cases, they can contribute to the management of an institution’s scientific research. In addition, it is recognized that each research field presents scientific collaboration practices grounded by its own guidelines, defined by the scientific policies to which it is submitted and by the individual or collective behavior of its researchers. A specialized institution in a knowledge domain also has its own characteristics of production and scientific collaboration and its management model can influence and characterize these forms of collaboration.

The profile of a scientific and institutional domain can be better portrayed by means of co-authorship analysis, given that it is characterized as a consolidated methodology for evidencing collaborative relationships in a certain domain. This argument is reinforced by Cronin et al. (2018), when they state that the most visible indicators to measure the trend for scientific collaboration are the national and international co-authoring rates, as the co-authoring data can be extracted from online bibliographic sources such as the Institute for Scientific Information (ISI) base (Cronin et al., 2018).

Posner (2001 apud Cronin et al., 2018) considers that academic work is increasingly teamwork. According to the author, several research have documented the growth of interinstitutional, interdisciplinary and intersectoral scientific publications conducted in collaboration in the second half of the 20th century.

Collaborative practices in science started to become institutionalized through the assignment of authorship in research, and then, in the 20th century, co-authorship analysis became consolidated as a collaborative scientific indicator in the advancement of science and its professionalization. It became an object of study and a methodology which is increasingly applied to measure collaboration in research projects in the contemporary science context (Hilário et al., 2020).

Co-authorship is one of the most tangible and well-documented forms of scientific collaboration, according to Glänzel and Schubert (2004). Almost every aspect of scientific collaboration networks can be reliably traced by means of co-authorship analysis, conducted by bibliometric procedures (Glänzel and Schubert, 2004).

Though the co-authorship practice is characterized as the most formally and widely known way of analyzing and evaluating scientific collaboration, it should be considered that it is not the only way to measure scientific collaboration and, like any indicator, it has limitations to measure collaborative reality according to each domain collaboration characteristics and object of study.

Table 1 illustrates the characteristics of co-authorship as a specific practice of scientific collaboration, as well as scientific collaboration in its most extensive way.

**TABLE 1:** Characteristics of co-authorship and collaboration in scientific research.

Co-authorship	Scientific collaboration	
a) Co-participation in the total or partial writing of the research results;	Collaboration on the scientific content	a) Indication of different analytical readings and perspectives
b) Data collection, organization and interpretation		b) Explanation of doubts and discussions about the subject studied
c) Analysis of results		c) Content validation, ensured by expertize in the research theme
d) Declaration of responsibility for the content		d) Oral narrative on essential historical contexts for the understanding of the events
e) Content review, orientation and validation		
	Collaboration on scientific practice	a) Orientations on technical, morphological and analytical aspects of the research
		b) Orientations on potential approaches that the research may receive
		c) Assistance with material management and collection
		d) Contribution with bureaucratic issues to make the research viable
		e) Space and/or material resources sharing, such as the use of laboratories
		f) Publishing assistance

Source: Hilário et al. (2018, p. 17), adapted by Hilário and Freitas (2020).

Scientific collaboration can be identified and analyzed by several documents that go well beyond publications, despite being often measured by means of co-authorships (Vanz and Stumpf, 2010). Furthermore, data from authors and co-authors are found to be readily accessible and interoperable, since they are available as metadata in databases.

In Table 2, elaborated by Hilário et al., 2020, it is indicated that the peculiarities of co-authorship practice lies in the fact that it is portrayed by the joint signature of scientific products, whether they are in their most usual format of articles or in research projects and technical-scientific reports.

**TABLE 2:** Methods and measurement units of collaborative activity in science.

Ways of measuring scientific collaborative activities		
**Analysis unit**	**Co-authorship**	**Collaboration**
Articles and/or scientific publications of other nature	- Joint signature (researcher, institution, country)	-Significant collaborations, expressed in the way of appreciations and financing
	- Number of authors	-Collaborations indicated as bibliographical references and in materials and methods
	- Order of authors	
	- Type of relationship between the authors	

Directory of research groups	- List of publications or co-authored research projects	- Interaction between researchers through participation in the same research group(s) (researcher, institution, country)
		- Thematic approximation among researchers, according to their performance in similar lines of research
		- Sharing of theoretical trends and lines of thought, highlighted in projects and publications
Institutional documents, such as forms, résumés, among others	- Co-authorship registrations in scientific documents (projects, reports) that are part of institutional documentary funds	- Records of academic genealogy relationships (mentor-mentee)
		- Registers of interinstitutional agreements
		- Records of application for research and other subsidies
		- Records of request for resources for visiting professors/researchers
		Records of participation in evaluation commissions

Source: Hilário et al. (2020).

In line with the portrayed approaches, it is considered that the study of collaborative relationships by co-authorship enables the characterization of author and institution competence domains, particularly when the emphasis is not on collaboration itself, but on the “researcher” element of analysis and its products which are generated under contextual and temporary conditions. The “researcher” element and its “scientific products” which derive from social and professional relations are generally bound to institutions. In this regard, it is considered that the study of these relationships and products can contribute to the definition of competence niches in research and the visualization of opportunities and gaps of knowledge to be exploited in the institutional scope.

Other dimensions to be exploited in the co-authorship measures of an institutional domain would be the potential associations between collaboration, productivity, visibility, impact and financial support of research. Price and Beaver (1966) identified in their studies a strong association between collaboration and productivity, in which the higher the collaboration, the greater will be the productivity.

The number of co-authors in a publication can be closely related to the degree of internationalization of a domain, in other words multiple authorship with international collaboration tends to provide more visibility and impact to research, as we can see here in this study. Oromaner (1975, apud Ajiferuke, 1988, p. 20) arguments that “single authored articles are somewhat less likely to have an impact than are multiple-authored articles.” Freitas et al. (2017) definition for impact and visibility is adopted. According to the authors, the impact of the articles is their recognition by the scientific community by means of the citations received. In contrast, visibility is understood as the range of dissemination of articles and their publishing journals, as determined by the coverage and scope of the index databases. In summary, visibility is the display that favors an article to be seen, accessed, read, cited and recommended by research pairs.

Similarly, the order of authorship, in other words, the positions held by the authors in a publication, contributes to the assessment of the collaboration degree of each co-author. Nevertheless, measuring this degree of collaboration is not an easy task, as each domain adopts a behavioral model. Authorship ordering can be, for instance: by alphabetical order, by contribution order, by reverse hierarchy, among other subjective criteria adopted in some authorship assignment cases (Hilário et al., 2020).

With the exception of the first author position as the main research collaborator, it is difficult to define degrees of collaboration for the remaining co-authors. In this regard, we agree with Zuckerman (1968) that the order of remaining co-authors does not always reflect the degree of each one’s contribution to the study. For this reason, this article adopted the identification of the first and last author with affiliation to the studied Institute, as the last author, in many cases, is the coordinator or leader of the research project, that is, the person who validates the study. Therefore, the behavior of the institutional scientific domain focused on the Biological Sciences was considered for this option.

Another interesting issue to be raised in the co-authorship framework is the association between research funding and multiple authorship. For Hefner (1981) financial support for research is closely associated with the number of people involved in the knowledge production for publication (in such a way as to consider authors and sub-authors).This association is particularly strong in the Biological Sciences and Chemistry, according to the author. But funding is not necessarily the cause that increases scientific collaboration, as it is likely that the more people involved in a research project, the greater will be the need for financial resources (Hefner, 1981). Another interesting finding is the positive relationship between the greater acceptance frequency of articles which were written under multiple authorship, as evidenced by Gordon (1980, apud Ajiferuke, 1988, p. 16) in the area of Astronomy.

Based upon the studies and trials mentioned in this theoretical framework, it is considered that the number of co-authors and their order enables us to measure the characteristics of a scientific or institutional domain, in agreement with the typical collaborative behavior of this domain. It is therefore assumed that the co-authorship study allows for the recognition of the role and leadership that these actors exert in the research of a domain and, for this reason, such analysis elements are contemplated in the results.

## Materials and Methods

This article adopts metric studies of information, more specifically co-authorship analysis to recognize INMA’s institutional collaboration network, its impact and scientific visibility throughout its historical trajectory as a public scientific research unit.

Initially, the mapping of INMA’s research collaborators was performed, both on institutional documents and by means of searches on the author indexes of the databases per institutional affiliation. Therefore, this mapping was carried out both from inside to outside and from outside to inside the Institute, for the recognition of its Universe of collaborating researchers. 93 researchers have been identified since 1984, and 41 (44%) of them have presented scientific production which are connected to INMA in the Scopus database since 1993. Those 41 collaborators in research (Supplementary Material S1)1 were characterized with respect to their types of ties with the Institute and the scientific production resulting from each one’s period of tie was recovered.

The types of ties were categorized into formal and informal. The identified formal ties were classified into temporary (scholarship holders) and permanent (public workers). Informal researchers, recognized as volunteers, were also classified as temporary.

The production for the period of each researcher’s relationship with the Institute was retrieved by means of the Scopus database. This corpus of articles was organized in Microsoft Excel spreadsheets, by the respective titles, with the following variables sequentially listed for each title: co-authors; type of authorship defined by the number of authors; type of ties of the authors with the INMA affiliation; number of citations received for the article; thematic prominence percentage of the article (Scopus indicator), impact index of the article (Scopus Field-Weighted Citation Impact - FWCI indicator); thematic topics of the article; international collaboration; partner countries; authors with INMA affiliation who held positions of first or last authors; and, finally, the number of authors affiliated to INMA in the same article was identified. The analytical table (Supplementary Material S2) made up by means of the identification of the mentioned elements allowed the generation of tables in Microsoft Excel. These variables and their intersections allowed us to assess who does the research at INMA and for INMA, which are the leaderships resulting from the collaborative networks, what is the role of these actors for INMA, what is the impact and visibility of the institutional research, how the type of relationships of the researchers influence the profile of the performed research, thus contributing to the creation of an institutional identity.

The co-authoring networks and keywords (indexed terms by Scopus database) were created by means of the VosViewer Software. Throughout the process of network creation, in order to clean up the data while avoiding ambiguity of terms and/or authors' names with spelling variations, two thesaurus were created in .txt format, one for co-authoring and one for indexed words. The word cloud was created with the free tool Word Clouds.2

## Results and Discussion

The National Institute of the Atlantic Forest (INMA) research body has been, predominantly, of temporary nature. The prevalence of temporary researchers interferes with the ways of defining institutional collaboration, which hinders its mapping, due to the dynamic nature of the human resources framework. Since it became a public institution in 1984, the Museum of Biology Professor Mello Leitão, currently INMA, has had a total of 77.5% scholarship holders, 7% of which are public workers and 15% are volunteer or informal researchers. Of these, 57% are male and 43% female. These numbers were estimated in 2020 for this survey and reflect the institutional context from 1984 to 2019. While 68% of the male researchers are included in the author indexes of Scopus and Web of Science databases, only 25% of the female researchers are included in these databases. This fact demonstrates the significant inequity of the presence of the genders in the scientific visibility bases as far as the science of the Institute is concerned.

With regards to the research corpus here analyzed (Supplementary Material S2), 138 affiliated articles to INMA were located in the Scopus database, authored and co-authored by 41 INMA research collaborators (44% of the total collaborators). Of these 41, only 24.3% are female researchers. The articles of the corpus, derived from the declared affiliation of their authors and/or co-authors with INMA are distributed over the course of 26 years, from 1993 to 2019. The co-authors of the articles representing INMA were categorized in accordance with their type of ties in Table 3.

**TABLE 3:** Distribution of INMA affiliated articles according to the types of institutional links of their research collaborators (1993–2019).

	Number of researchers	Articles	Total citations	Average citations
Scholarship holders	21	87	337	2.4
Public workers	6	17	197	15.4
Volunteers	17	108	1.521	17.1

Note: The citation average for each tie category was calculated from the average citation of each collaborator for the publications he or she produced when affiliated to INMA. If a single author obtained more than one type of link in different periods, his or her average was considered for the different categories. The scientific production coverage for the authors of the INMA collaborative network occurred from 1993 to 2019 at the Scopus base, but the survey of institutional collaborators was carried out from 1984 onwards.

Source: Developed by the authors.

It can be noted that the greatest number of articles, as well as the greatest citation impact are derived from authors who are distinguished by informal ties of volunteer researchers.

Although the analyzed corpus is quantitatively small to express an institutional behavioral pattern, the body of researchers with formal ties that most collaborated under the productivity aspect of the articles were scholarship holders, although their publications presented low citation impact (Table 3). Volunteer designated researchers have the highest citation average, close to the average of the public workers.

It is likely that the drivers for research by volunteer researchers, without formal institutional ties, would be: the research potential offered by the Institute in terms of favorable and productive conditions and space for studies associated with the Atlantic Forest; personal and professional relationships that potentially connect them to the few members who are formally linked to the Institute; and the existence of scientific collections which are allocated at INMA.

It is important to highlight that a researcher may be included in more than one type of bond category, for example, he may have been a volunteer and then become a public worker or a scholarship holder. This is a key explanation for a better understanding of Table 3. Therefore, the consigned production to a single researcher may have been accounted for in one, two or more category ties so that a compatible result with the institutional research reality may be attained.

It is noteworthy that 27% of the corpus of articles which were analyzed presents international collaboration with the highlighted countries in Table 4, which can be regarded as a high share of internationalization for a low-profile institutional domain in the national scientific field. Referenced studies on the theoretical framework of the article corroborate that the establishment of international partnerships can increase the visibility of publications. On the other hand, the wider the scope of scientific activity and recognition of an institutional or scientific domain at a national level, the more it will be expected that it establishes collaborative relationships at a national level.

**TABLE 4:** Main countries contributing to the research of the corpus (1993–2019).

Collaborating countries	Articles
United States	22
United kingdom	8
Argentina	4
Germany	3
Colombia	3

Source: Developed by the authors.

It is observed that the international collaboration is more significant with the five countries highlighted, most intensely with the United States. It is also suggested that this is significant for a research institute which is still incipient and undergoing institutional restructuring, such as INMA.

The collaboration with the United States is particularly highlighted in herpetology, a zoology specialty that studies amphibians and reptiles. The prominent topics arising from the partnership with that country are *Leptodactylidae, Hylidae, Anura; Leptodactylidae, Squamata and Teiidae, Lizards*; followed by the Primatology subarea with the topics: *Adelina*; Yellow Fever Virus, *Haemagogus; Flaviviridae, Alouatta, Atelidae and Primates*. With a less dense participation in the partnership with the United States, entomology consigned to zoology and botany are highlighted. Herpetology studies are distinguished in the partnership with Germany.

The international collaboration with the United Kingdom is especially due to the domain of mastozoology, with subjects such as:*Didelphimorphia, Opossums, Marsupia; Myrmecophaga, Tridactyla, Sloths, Pilosa, Porcupines; Hystricidae, Erethizontidae*; in primatology with *Alouatta; Atelidae, Primates* and, finally, in Botany with *Myrtaceae, Myrcia, Eugenia and Begonia, Petermannia.*

Regarding the collaboration with Colombia, the topics *Aristolochiaceae, Perianth, Asarumse*are highlighted. The collaboration with *Argentina* covers the following topics: Coastal Plains, Holocene, Sea-Level Change; Biological Soil Crusts*; Lichen Crusts, Microcoleus and Begonia, Petermannia, Sect.*

The botany sub-area has a greater range and dispersion of collaborating countries in the analyzed corpus. Therefore, this collaboration is apparently less dense than in the zoology sub-area, where there is a higher prevalence of collaborative occurrences with the countries mentioned in Table 4. Moreover, zoology accounts for 65.9% of the total analyzed corpus, with botany accounting for 30.4% of the total corpus.

Concerning the types of authorship, and with respect to the number of authors, 60.8% of the corpus is consigned to individual, two and/or three authors, with a higher percentage for dual authorship (26.1%) (Table 5; Supplementary Material S2). It is suggested that multiple authorship in the highest incidence would have the potential to provide more visibility to publications.

**TABLE 5:** Types of authorship identified in the institutional production corpus (1993–2019).

Types of authorship	Number of articles	(%)
Individual	14	10.1
Two authors	36	26.1
Three authors	34	24.6
Four authors	15	10.9
Five authors	18	13.0
Multiple	21	15.2
Total	138	100,0

Source: Developed by the authors.

In contrast, the predominance of double and triple authorship in most of the articles in the corpus presenting the first and last authors with affiliation to INMA may suggest the possibility that the Institute may head or lead the research by aggregating partnerships to its publications (Table 5). However, the merits of the impact and visibility of publications in individual authorship of researchers with the affiliation of the Institute are not excluded, as the literature suggests that the probability of accepting a research in a simple (individual) authorship is lower than in multiple authorship (Gordon 1980, apud; Ajiferuke, 1988, p. 16). It should be highlighted that 57% of individual authorships are related to botany domain (Supplementary Material S2).

Based upon these authorship variables, the institutional research is better consolidated in some zoology specialties, which will be better portrayed throughout the article.

Regarding the presence of authors with INMA affiliation in first and last positions, it can be noted that in 64.4% (89 articles) the first and last authors are co-authors who declare affiliation with INMA (Supplementary Material S2). This result indicates a good leadership level in the research being conducted by the Institute, particularly when this information is allied to the fact that 17.3% of the articles have more than one co-author linked to INMA.

On the thematic relevance of the articles of the corpus for international science, there is the indicator called thematic prominence (made available by the Scopus database), which was identified for each article and intends to show a topic’s current dynamics in international science. This indicator is calculated by means of three metrics for documents that have been grouped into a topic, namely: citation count, Scopus views and average CiteScore (Scopus citation average).

Considering the fact that 77.5% (107) of the articles in the corpus consigned to INMA work with thematic topics that have a prominence above 70% (Column H in Supplementary Material S2), it is thus proven the high relevance of the articles produced on the themes addressed within the contemporary international context. Topic Prominence in Scopus database provides a picture of overall research performance, and insight into the momentum or level of activity of particular Topics.3 The higher the number of this indicator, more important the theme is in the current international context.

**FIGURE 1:**
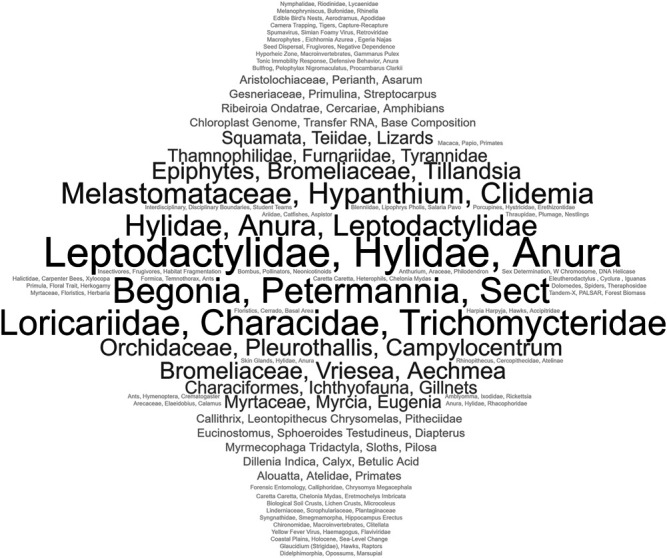
Cloud of topics of higher incidence and high prominence of the analyzed institutional production (1993–2019).

The word cloud in Figure 1 presents the topics that have been most recurrent in the analyzed articles and that, at the same time, distinguished themselves due to the high rate of thematic prominence in the Scopus base.

The groups of topics related to the amphibian family *Hylidae* and *Leptocdactylidae* of the *Anura*Order are distinguished on the cloud, with 75.5% and 85.4% thematic prominence, respectively; the fish family called *Loricariidae*, with 89% prominence, contemplating the popularly called catfish and shellfish; the botanical family *Melastomatacea*, belonging to the *Myrtales* order with 78.7% prominence, and the topics named *Bromeliacea* with 74.2% prominence and *Epifithi* with 85% prominence.

Research in the field of zoology is distinguished owing to the greater impact of citation, given that, among the ten articles with the greatest citation impact of the corpus, 80% is in the field of zoology. In contrast, research in the field of botany are predominantly in individual or dual authorship, with a less significant citation impact, as well as a smaller number of articles as already portrayed (Supplementary Material S2).

In order to complement institutional production profile, the network of thematic co-occurrence is observed by the terms indexed in the Scopus base for the INMA corpus. The network reflects the Institute’s research in all its historical presence at Scopus.

**FIGURE 2:**
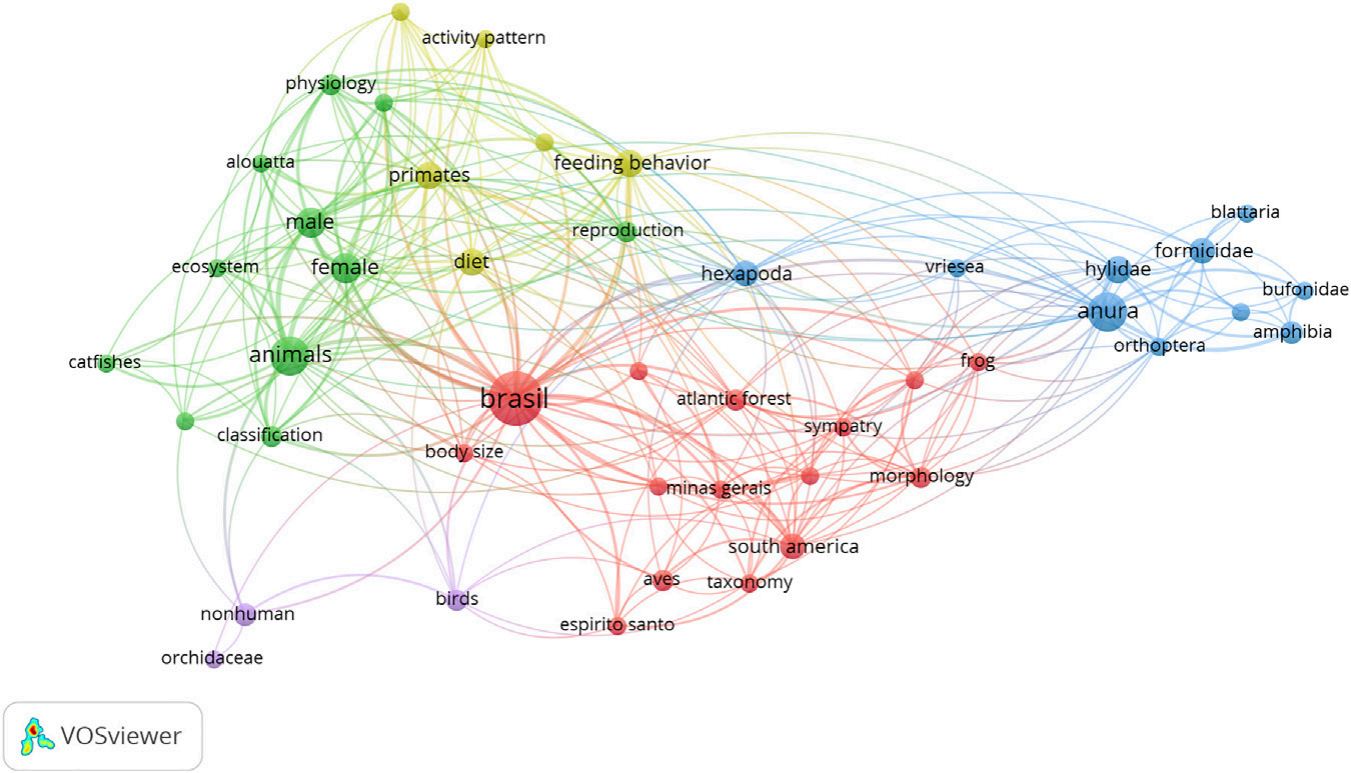
Co-occurrence between the analyzed institutional production terms (1993–2019).

The terms presented in Figure 2 have been applied at least three times and, after their standardization, they totaled 51 terms. Five clusters are observed, distinguished by colors, which establish relationships in the INMA’s scientific institutional domain. Among the denoted themes in the network, the following are evidenced: studies related to the amphibian and reptile morphology; reproduction and feeding habits of mammals and, especially, primates; studies on the endemic birds of the biome; studies on insects, as represented by the term Hexapoda; and, also, related studies to the taxonomic classification of flora and fauna.

Of the five groups, the blue cluster of herpetology stands out. The network becomes more concentrated in the amphibian/anura theme, which also brings together the highest percentage of international collaboration, followed by the field of primatology, consigned to zoology, and finally botany.

**FIGURE 3:**
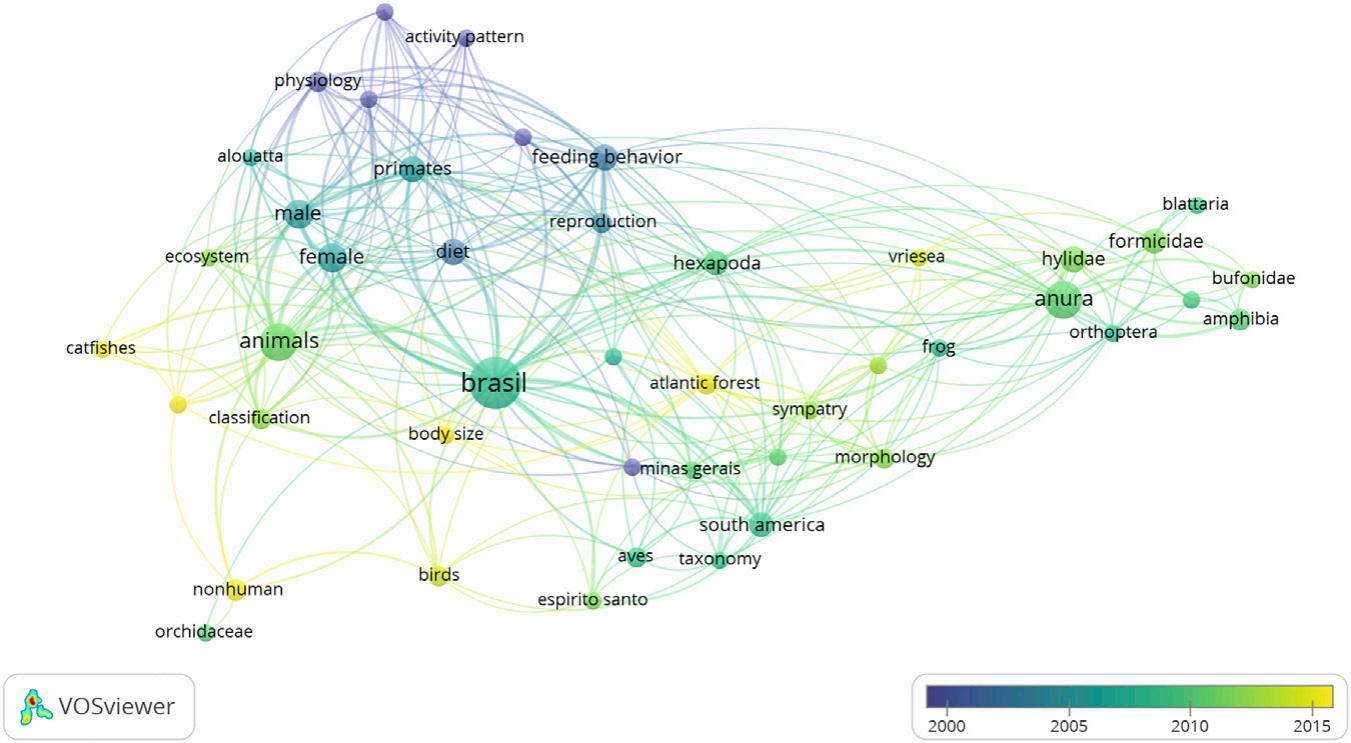
Co-occurrence between the indexed terms of the analyzed institutional production and their incidence every five years (1993–2019).

When the distribution of terms is observed over the course of the 20 years (Figure 3), the prevalence of the study of mammals and, especially, primates between the years 2000 and 2005 is more significant. Between the years 2005 and 2010 amphibians stand out as the object of the articles. From 2010 to 2015, the domain of ichthyology (fish) as well as ornithology (birds) become more evident in the analyzed publications. The latter sub-area stands out both in terms of green and yellow for the last five years (2015–2020) of article production.

The distribution of these themes follows, of course, the specialties and lines of action of researchers affiliated to INMA in these periods. As a result, these researchers shared with the Institute their intellectual capital (knowledge) and their social capital (networks of co-authors and inter-institutional and inter-country partnerships). The Institute, in turn, provided the fundamental conditions for the publication development, with financial, operational, technical and human contributions, accomplished by means of research grants, infrastructure, material resources, auxiliary human resources and especially, in physical space, locus of knowledge.

**FIGURE 4:**
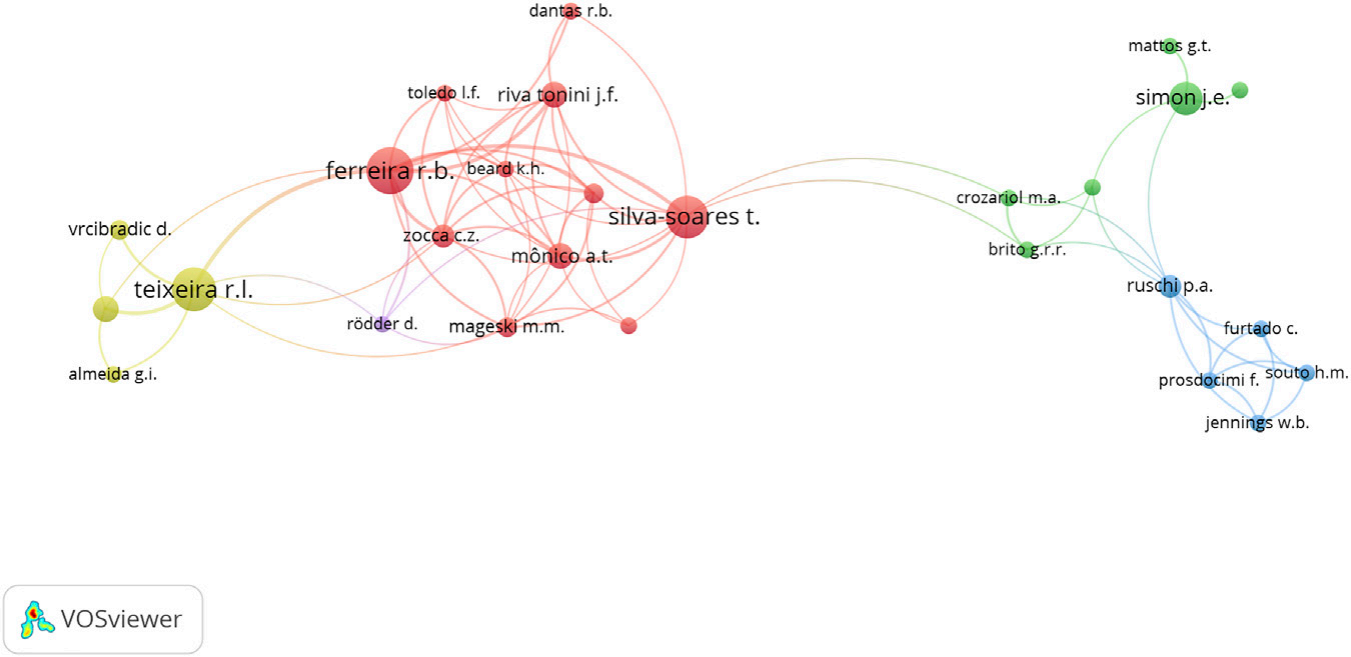
Network of co-authors that make up INMA’s collaborative scientific research identity (1993–2019).

The represented reality of the highlighted themes is corroborated in the established co-authorship network. In order to develop INMA’s co-authoring network, the criteria was that the author had at least two articles with INMA affiliation and had established at least one co-authoring relationship with other researchers. Articles with more than 25 authors were excluded, as well as articles of individual authorship. The network of co-authors in Figure 4 evidenced scholarship holders and volunteers due to the greater number of articles consigned to them.

Figure 4 shows the five collaboration clusters that are related at the institutional level and distinguished by colors. The actor who most establishes the intermediation of the network is Silva-Soares, whose relationships allow the interlocution of its red cluster (herpetology) with the green cluster (ornithology). Regarding the density of the network, the concentration of articles and relationships is in four actors: Teixeira (herpetology/ichthyology), Ferreira (herpetology), Silva-Soares (herpetology) and Simon (ornithology), which are considered the main actors in the institutional network. They represent the categories of volunteers and/or scholarship holders.

Ferreira is the main actor of the red group (herpetology), which makes the connection with the yellow (ichthyology). There was a remarkable partnership between Ferreira and Silva-Soares from the same group, and between Ferreira and Teixeira from the yellow group.

It is noteworthy that the public workers and volunteers, who have the highest citation impact, do not appear on the network. This occurs owing to the smaller number of articles published by them within INMA’s membership and due to their individual authorship publications. Nonetheless, they can play an important role in the history of the Institute, either as managers, or as technicians, or as volunteer researchers.

**FIGURE 5:**
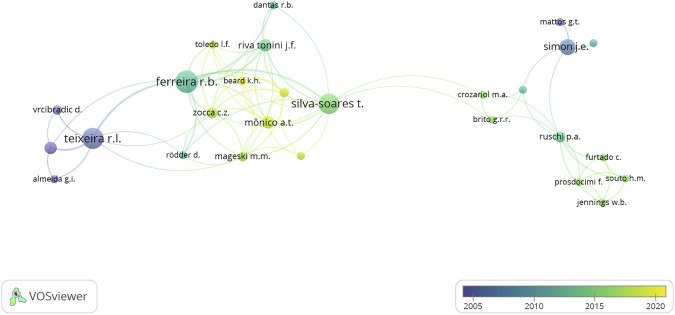
Network of co-authors that make up the INMA’s collaborative identity of scientific research and its incidences every five years (1993–2019).

Figure 5 shows the incidence of collaborations as per time frame of every five years. From 2005 to 2010, Teixeira and Simon’s group emerged with research related to fish and birds. Between 2010 and 2015, the herpetology group gained prominence.

Finally, the herpetology group has continued with significant participation from 2015 to 2020, however consigned to the production of scholarship holders Zocca and Mônico. In general, it is considered that the main actors in the network with higher production density and also with a higher degree of intermediation are scholarship holders and volunteers with temporary ties.

## Conclusion

This study allowed establishing the profile of INMA’s collaboration and institutional scientific production, considering its historical trajectory as a public research unit. This profile appears to be atypical for a Brazilian science and technology research unit, since it presents a significant number of researchers who are considered temporary, either as scholarship holders or as volunteer researchers.

It is also considered that the institutional research scene reflects the social and political context in which INMA was created as a continuation of the Museum of Biology Professor Mello Leitão, whose transformation into a research unit with the mission of developing science within the Atlantic Forest is marked by scarcity of infrastructure, human, financial and material resources. An example related to the referred scarcity would be, for example, the insignificant number of permanent researchers in the Institution. However, the indicators obtained showed that the science produced by INMA, in addition to having national and international relevance, reflects the fulfillment of its institutional competence related to the research development.

The prominent areas constituting the profile of institutional research were Zoology and Botany. Zoology, besides its greater impact, accounts for more than half of the corpus production (65.9%). On the other hand, botany is responsible for 30.4% of the corpus, with its dispersed international cooperation in a broad variety of countries. Macro-level collaborative relations identified in the corpus are more intense with the United States, covering 16% of the total corpus of articles in cooperation with that country. The scientific production highlighted in these fields corroborates the importance of the biological collections allocated at the Institute. The zoological collection, with 42,443 records (Specieslink, 2020), is composed mainly of amphibians, fishes and birds, which converges with the results of the presented bibliometric analysis. The collection of plants of the Herbarium MBML has 54,325 records (Specieslink, 2020), with emphasis on orchids, also evidenced in the results presented (orchidaceae- Figure 2).

In addition, the identification of the order of authorship in articles and the participation of more than one author of the Institution in a single document indicates research leadership by the Institute in the analyzed corpus, which ends up leading the research co-authored with other institutions and researchers.

The methodology employed to portray this domain has made it possible to recognize that the scientific production made by temporary collaborators also contributes to the expansion of the Institute’s visibility in global science and to its support as a research center.

The potential of co-authorship analysis and metric studies of information for the creation of a scientific and collaborative identity in this institutional domain was also shown. In short, the profile of collaboration and research found contributes with the domain analysis under complementary dimensions such as the historical one, whose differentiation lies in the possibility of transcending the registered documentation, which can be measured by bibliometric procedures, in order to rescue the constituent narratives of the institutional consolidation process.

## Data Availability Statement

The original contributions presented in the study are included in the article/Supplementary Material, further inquiries can be directed to the corresponding author.

## Author Contributions

JF conceived and conceptualized the study; identified, collected and organized the information on the institution’s collaborating actors, established the analysis elements and the variables to be targeted in the indicators and conducted the analysis and discussion of the results. FR collected and organized the production, collaboration and impact bibliometric data of the analysis corpus, developed the authors' thesaurus and terms for the network formation, proceeded to structure the morphological and technical aspects of the publication. All authors contributed to the manuscript reviews by having read and approved the submitted version.

## Funding

Scholarship from the Institutional Training Program of the National Council for Scientific and Technological Development (CNPq) of the National Institute of the Atlantic Forest (INMA). Process no: 302392/2020-3.

## Conflict of Interest

The authors declare that the research was conducted in the absence of any commercial or financial relationships that could be construed as a potential conflict of interest.
