# Supplementary material for: Scientific Collaboration at National Institute of the Atlantic Forest (Brazil) on Scopus Database: Analysis of Institutional Domain
Source: Front Res Metr Anal. 2020 Dec 17;5:601442. doi: 10.3389/frma.2020.601442 (PMC8025981; doi:10.3389/frma.2020.601442)
Supplement: Supplementary file 1 [file datasheet1.pdf]

Supplementary Material 01

1 Supplementary Tables

Volunteers

Authors	Articles	Citations	Citation Average
Volunteers 01	1	19	19
Volunteers 02	6	313	52,2
Volunteers 03	1	2	2
Volunteers 04	14	113	8,1
Volunteers 05	3	19	6,3
Volunteers 06	30	143	4,8
Volunteers 07	1	1	1
Volunteers 08	11	90	8,2
Volunteers 09	7	57	8,1
Volunteers 10	4	119	29,8
Volunteers 11	2	6	3
Volunteers 12	4	4	1
Volunteers 13	1	46	46
Volunteers 14	2	48	24
Volunteers 15	6	256	42,7
Volunteers 16	14	270	19,3
Volunteers 17	1	15	15
**Total**	**108**	**1521**	**17,1**

Scholarship holders

Authors	Articles	Citations	Citation Average
Scholarship holders 01	1	4	4
Scholarship holders 02	2	0	0
Scholarship holders 03	1	2	2
Scholarship holders 04	1	0	0
Scholarship holders 05	1	3	3
Scholarship holders 06	1	2	2
Scholarship holders 07	1	0	0
Scholarship holders 08	30	143	4,8
Scholarship holders 09	1	1	1
Scholarship holders 10	1	1	1
Scholarship holders 11	5	11	2,2
Scholarship holders 12	1	0	0
Scholarship holders 13	2	3	1,5
Scholarship holders 14	15	113	7,5
Scholarship holders 15	3	1	0,3
Scholarship holders 16	1	0	0
Scholarship holders 17	10	20	2
Scholarship holders 18	3	1	0,3
Scholarship holders 19	1	0	0
Scholarship holders 20	1	15	15
Scholarship holders 21	5	17	3,4
**Total**	**87**	**337**	**2,4**

Public workers

Authors	Articles	Citations	Citation Average
Public workers 01	2	11	5,5
Public workers 02	1	46	46
Public workers 03	3	2	0,7
Public workers 04	1	8	8
Public workers 05	7	57	8,1
Public workers 06	3	73	24,3
**Total**	**17**	**197**	**15,4**
